# Retrieving challenging vessel connections in retinal images by line co-occurrence statistics

**DOI:** 10.1007/s00422-017-0718-x

**Published:** 2017-05-09

**Authors:** Samaneh Abbasi-Sureshjani, Jiong Zhang, Remco Duits, Bart ter Haar Romeny

**Affiliations:** 1 Department of Biomedical Engineering, Eindhoven University of Technology, P.O. Box 513, 5600 MB Eindhoven, The Netherlands; 2 Department of Mathematics and Computer Science, Eindhoven University of Technology, P.O. Box 513, 5600 MB Eindhoven, The Netherlands; 3 Department of Biomedical and Information Engineering, Northeastern University, 500 Zhihui Street, Shenyang, 110167 China

**Keywords:** Curvilinear structures, Line co-occurrences, Cortical connectivity, Contextual affinity matrix, Spectral clustering, Perceptual grouping

## Abstract

Natural images contain often curvilinear structures, which might be disconnected, or partly occluded. Recovering the missing connection of disconnected structures is an open issue and needs appropriate geometric reasoning. We propose to find line co-occurrence statistics from the centerlines of blood vessels in retinal images and show its remarkable similarity to a well-known probabilistic model for the connectivity pattern in the primary visual cortex. Furthermore, the probabilistic model is trained from the data via statistics and used for automated grouping of interrupted vessels in a spectral clustering based approach. Several challenging image patches are investigated around junction points, where successful results indicate the perfect match of the trained model to the profiles of blood vessels in retinal images. Also, comparisons among several statistical models obtained from different datasets reveal their high similarity, i.e., they are independent of the dataset. On top of that the best approximation of the statistical model with the symmetrized extension of the probabilistic model on the projective line bundle is found with a least square error smaller than $2\%$. Apparently, the direction process on the projective line bundle is a good continuation model for vessels in retinal images.

## Introduction

### Tracking curvilinear structures

Tree-like structures such as the retinal vasculature, corneal nerve fibers and roads from aerial photographs for cartography are widely studied both in quantitative computer-aided diagnosis systems in large-scale screening programs, and high-volume industrial settings. Delineation of curvilinear structures in these images is essential for investigating their characteristics. For instance, several studies highlighted the importance of using quantitative measurements of morphological and geometrical properties of blood vessels in retinal images for early diagnosis and prognosis of several diseases such as hypertension and diabetic retinopathy (e.g., Chapman et al. 2002; Smith et al. 2004).

Despite all improvements in the segmentation of curvilinear structures in two-dimensional images, the proposed methods often present limitations when two or more structures branch or cross, or when there are areas with missing information or interruptions (Fraz et al. 2012). Consequently, several tracking-based techniques provided solutions for preserving the connections in tree-shaped networks (e.g., Türetken et al. 2012; Cheng et al. 2014; Bekkers et al. 2014; Estrada et al. 2015; Hu et al. 2015; De et al. 2016). One of the common approaches has been to manually design cost functions, which penalized abrupt changes of the contextual features such as orientation, width and color. These costs were used in later stages in optimization or graph theory-based techniques for constructing the full retinal vasculature network. In these methods, not only the cost functions were designed manually and depended on existing topological structures, but also tracing errors were often created due to the use of imperfect pixel-based vessel segmentations and skeletons that do not guarantee the connections among pixels belonging to one vessel.

**Fig. 1:**
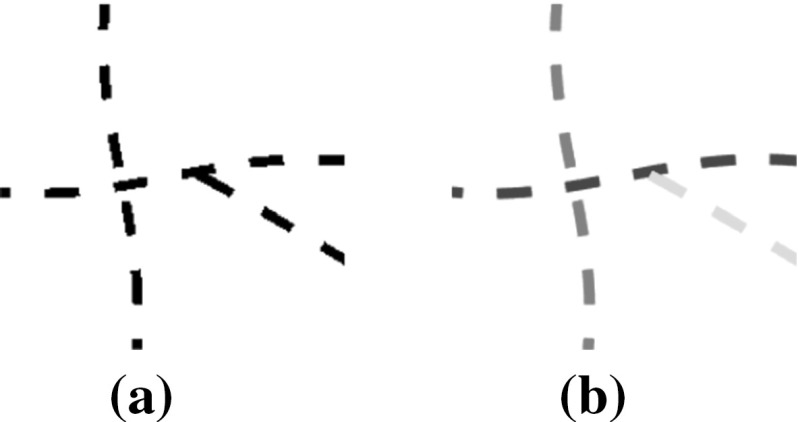
**a** A sample image with interrupted segments and **b** the salient units identified by our perceptual system

### Geometry of primary visual cortex

The human visual system is capable of interpreting visual scenes and of completing disconnected contours among interrupted segments, following the Gestalt law of good continuation (Wertheimer 1938). Figure 1 represents a sample interrupted phantom image (Fig. 1a) and the units detected by our perceptual system in different colors (Fig. 1b). Inspired by this capability, a new method was proposed by Favali et al. (2016) for resolving the missing and complex connections among blood vessels at junction points in retinal images. In this approach, first the image is lifted to the coupled space of positions and orientations using the so-called orientation score transformation introduced by Duits et al. (2007b). The multi-orientation score is augmented with a contextual affinity matrix inspired by the long-range contextual connections between the multi-orientation pinwheel structures discovered in the primary visual cortex (V1) (Hubel and Wiesel 1962; Bosking et al. 1997). The affinities in this matrix are augmented by another similarity measurement of the feature of intensity and further processed in a spectral clustering step, which resulted in separate groups each representing one individual blood vessel.

The cortical connectivity representing the contextual connections in V1 can be modeled as the fundamental solution of the time-independent Fokker–Planck (FP) equation for Mumford’s direction process (Mumford 1994; Williams and Jacobs 1997; August and Zucker 2003; Citti and Sarti 2006; Duits and Almsick 2008). Another closely related model for perceptual grouping of local orientations is a hypo-elliptic Brownian motion, whose FP equation describes hypo-elliptic diffusion without convection for the generator (Citti and Sarti 2006; Duits and Franken 2009; Agrachev et al. 2009). These models explain well the notion of association fields introduced by Field et al. (1993) as a model for contour integration by the human visual system for image perception. There exist various numerical approximations, and exact solutions. For a recent overview and detailed comparison of all the solutions, see Zhang et al. (2016b) and references therein. Based on this study, the Fourier-based technique (Duits and Almsick 2008) is the best approximation of the exact solution, having the smallest error both in the spatial and Fourier domains. The second best approximation is provided by the stochastic method based on the Monte Carlo simulation (Robert and Casella 2005). The stochastic solution was used by Favali et al. (2016).

### Edge statistics in natural images

Second- and higher-order edge statistics are commonly used for representing the mutual relation between connected edges in natural images. Several studies (e.g., August and Zucker 2000; Geisler 2008; Sanguinetti et al. 2010; Perrinet and Bednar 2015) investigated the statistics of the edges in our surrounding environment and their relation to the adaptation of connectivity patterns in our perceptual system. These studies showed that the individual edges are dependent on each other, and the strongest characteristic determining their connections is their co-circularity relation. In the work by August and Zucker (2000), the edge statistics for several types of image categories were measured and it was shown that the resulting patterns were dependent on the structures available in the images. In a recent study by Perrinet and Bednar (2015), these low-level edge statistics were used for a high-level judgmental task successfully. Moreover, Sanguinetti et al. (2010) showed that there is a close relation between the statistics of edge co-occurrence and the probabilistic, geometric model of the cortical connectivity in V1.

### Our proposed method

We propose to train the probabilistic connectivity kernel using the line co-occurrence statistics on the lifted space of positions and ($\pi$-periodic) orientations $\mathbb {R}^2\times \textit{P}^1$ extracted directly from the retinal images. To this end, we make an adaptation of the direction process to the projective line bundles in $\mathbb {R}^2\times \textit{P}^1$ instead of ${\mathbb {R}}^2\times S^1$ (the space of positions and $2\pi$-periodic orientations), as this extension is necessary for comparison of the probabilistic model to the statistical co-occurrences. By comparing the statistical kernel to the symmetrized probability kernel, its best approximation resulting in the least square error is found. In fact, we show the relation between the probabilistic model of cortical connectivity and the edge statistics in our retinal imaging application is even closer when including both symmetrization and a projective line bundle structure. The dependency of the parameters of the statistical kernel with respect to the dataset is also investigated using different retinal image datasets, varying their resolutions and pixel sizes.

Finally, we show the application of this trained model in retrieving the vessel connections at locations with complex structures in retinal images. To this end an affinity matrix is created based on this statistical model and the similarities among vessel intensities and is analyzed in a self-tuning spectral clustering technique (Zelnik-Manor and Perona 2004), which does not need any parameter tuning and manual thresholding of the eigenvalues. It automatically determines the number of salient groups in the image by rotating the eigenvectors to create the maximally sparse representation and by minimizing a clustering cost defined accordingly.

Summarizing, we demonstrate the following points in this article:

1. The statistical line co-occurrence kernel learned from retinal images matches remarkably well our symmetrized extension of the probabilistic model on the projective line bundle;
2. The statistical kernels do not change significantly over different datasets and are reproducible;
3. The low-level line statistics are successfully used to perform the high-level task of grouping of the interrupted blood vessels in the retinal images automatically;
4. Mumford’s direction process is a very good stochastic model for connecting interrupted vessels in segmented retinal images.

### Paper structure

The rest of the article is structured as follows. In Sect. 2, the steps for deriving the line co-occurrences from retinal images and the theoretical details about modeling the cortical connectivity are described. In Sect. 3, after introducing the datasets, the resulting line co-occurrences are presented and compared against each other quantitatively and qualitatively. The best probability model approximating each kernel is presented afterward. Application of the statistical kernel in retinal image analysis is presented at the end of the section. Finally, the results are discussed and the paper is concluded in Sect. 4.

## Methodology

In this section, in addition to introducing the steps for extracting the line co-occurrences from retinal images, a numerical model of the connectivity kernel is proposed.

### Line co-occurrence

In order to extract the line co-occurrences from retinal images, a similar approach as the method of Sanguinetti et al. (2010) is used. However, there are some differences. We only use retinal images, which include multiple elongated structures: the vessels; the vessel centerlines have been used instead of the edges (the resulting kernel is called *line co-occurrences* rather than *edge co-occurrences*), and no line polarity has been taken into account; the orientation score transformation has been used to find the orientation information at each point.

Recall that the projective circle $\textit{P}^1$ is obtained from the normal circle $\textit{S}^1$ by identifying antipodal points. The orientation score (OS) transform ${\mathbb {R}}^2\rightarrow {\mathbb {R}}^2\times \textit{P}^1$ is obtained by correlating the input image *f* with rotated isotropic (bi-directional) cake wavelets $\psi$ (Duits and Franken 2009; Bekkers et al. 2014) in $n_\theta$ directions ($\theta \in [-\pi /2,\pi /2-\pi /n_\theta ]$) as:

1 $$\begin{aligned} \begin{aligned} U_f(\mathbf {x},\theta )&= ~\left( \overline{\mathbf {R}_\theta (\psi )} \star f\right) (\mathbf {x}) \\&= \int _{{\mathbb {R}}^2} \overline{\psi \left( \mathbf {R}^{-1}_\theta (\mathbf {y}-\mathbf {x})\right) }f(\mathbf {y})\mathrm {d}\mathbf {y} \end{aligned} \end{aligned}$$

where $\mathbf {R}_\theta$ is the 2D counter-clockwise rotation matrix, the overline denotes the complex conjugate and $\star$ denotes the correlation (Duits et al. 2007b). The cake wavelets are quadratic anisotropic filters similar to the Gabor wavelets, but unlike them, their summed Fourier transformations cover the entire frequency domain (up to the Nyquist frequency), making them spatially scale-independent. Moreover, the invertibility property of orientation score transformation prevents information loss during the transformation (Duits et al. 2007a, b). Therefore, the cake wavelets are appropriate choice for lifting the crossing and bifurcating blood vessels regardless of their varying widths.

The proposed method for finding the line statistics of the images of a dataset $\textit{S} = \{I_1,I_2,\dots ,I_n\}$, where $I_i\in {\mathbb {R}}^2$ is the *i*th retinal image, is explained step by step in Algorithm 1. The initial step is to create a set of interest vessel positions and orientations for each image. To obtain the vessel pixel locations the vessel ground truth is used, and the binary vessel centerlines ($\textit{I}_{c,i},i=1,\dots ,n$) are extracted in a standard morphological thinning approach (Lam et al. 1992). So if a pixel at location (*x*, *y*) belongs to a centerline, then $\textit{I}_{c,i}(x,y)=1$, otherwise $\textit{I}_{c,i}(x,y)=0$. If the ground truth is not available, then the vessel segmentation is obtained using one of the state-of-the-art techniques (e.g., Zhang et al. 2016a). The orientations at interest centerline positions are obtained by lifting the image using the OS transform (Step 2), and finding the angles with the maximum response at these locations [$\theta _{m_i}(\mathbf {x})$ in Step 3]. It is worth mentioning that only the real part of the OS has been considered which acts as a ridge detector on the Gaussian profiles of blood vessels. Besides, the blood vessels in retinal images are darker compared to the background. As a result, they get negative responses (large absolute values) in this transformation. The negative sign used at Step 3 compensates for that. These centerline locations and their corresponding dominant orientations are later used in Step 4 to create a set of interest points called $\textit{L}_i$ for each image.

**Fig. 2:**
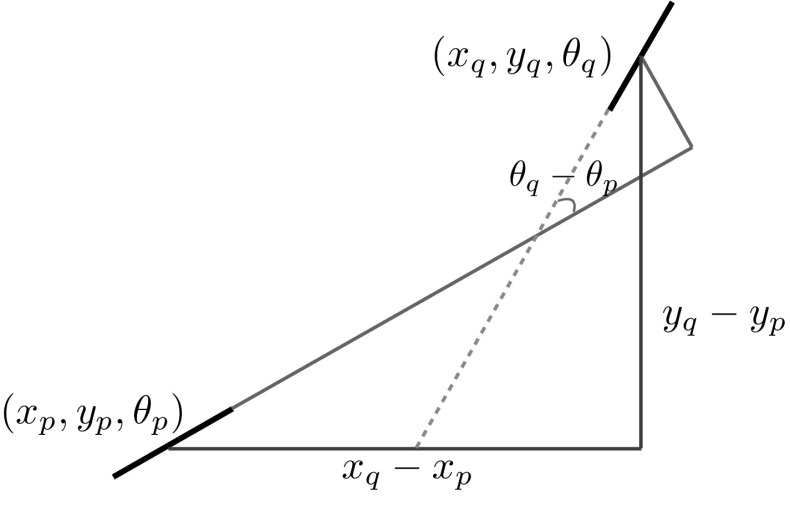
A sample pair of edges with positions and orientations of $(\textit{x}_p,\textit{y}_p,\theta _p)$ and $(x_q,y_q,\theta _q)$. The relative positions and orientations are depicted. Adapted from (Sanguinetti et al. 2010, Fig. 1)

In the next step, pairs of interest points located at less than a certain distance ($\textit{d}$) from each other are used to create a difference set $\textit{S}_i^\textit{d}$ considering the translation-invariance property (see Step 5 and Fig. 2). In order to make the set rotation invariant, the relative positions are rotated with respect to the relative orientations and the shift-twist difference set $\textit{Q}_i^\textit{d}$ is created in Step 6.

By counting the number of occurrences of relative positions and orientations in Step 7 the statistical kernel per image is created. The statistical kernel of the entire dataset is obtained by accumulating the individual kernels of all the images in set *S*. Finally, the kernel is $l_1$-normalized and called the data-driven or statistical kernel (Step 8). The final dimension of this kernel is $(2\textit{d}+1) \times (2\textit{d}+1) \times n_\theta$. The parameter *d* is selected heuristically during experiments.

In another approach, the artery/vein (AV) labels of vessels and the fact that arteries are not directly connected to veins (Sherwood 2012) are taken into account. By knowing these labels, the vessel profiles for arteries and veins are separated and their line co-occurrences are calculated individually ($K_{i,\mathrm A}^\mathrm{stat}$ and $K_{i,\mathrm V}^\mathrm{stat}$), using the similar steps as described before. Finally, the artery and vein histograms are added to each other to find the final histogram for each retinal image ($K_{i}^\mathrm{stat}= K_{i,\mathrm A}^\mathrm{stat}+K_{i,\mathrm V}^\mathrm{stat}$). This is a more accurate assumption about the connections among vessel centerlines; however, it is only possible to use this setup if the AV labels are available. More details about datasets, parameter settings and results are given in Sect. 3.
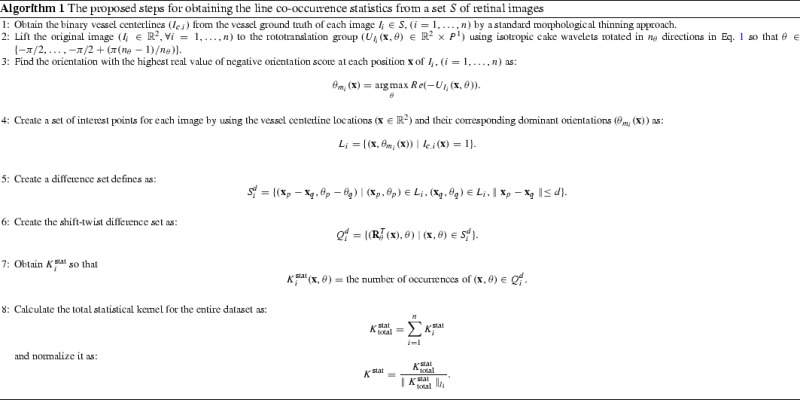


### Cortical connectivity in $\mathbb {R}^2\times \textit{P}^1$

Considering Mumford’s direction process in the differential structure of the sub-Riemannian SE(2) group, the fundamental solution of the FP equation represents the probability of having a contour at a certain position and orientation, starting from a reference position and orientation. In order to model the cortical connectivity kernel in ${\mathbb {R}}^2\times P^1$ (projective line bundle), we propose to create the connectivity kernel by adding the solutions of the FP equation in forward and backward directions in ${\mathbb {R}}^2\times S^1$ (for symmetrization) and the $\pi$-shifted solutions (for taking into account the final periodicity). Therefore, the connection probability between two points in ${\mathbb {R}}^2\times P^1$ is obtained by:

2 $$\begin{aligned}&k^\mathrm{{prob}}\left( (\mathbf {x},\theta ),(\mathbf {x'},\theta ')\right) \nonumber \\&\quad =\frac{1}{4} \bigg (\varGamma \Big ( (\mathbf {x},\theta ),(\mathbf {x'},\theta ') \Big ) + \varGamma \Big ((\mathbf {x'},\theta '),(\mathbf {x},\theta ) \Big ) \nonumber \\&\qquad + \varGamma \Big ((\mathbf {x},\theta +\pi ),(\mathbf {x'},\theta ')\Big ) + \varGamma \Big ((\mathbf {x'},\theta '+\pi ),(\mathbf {x},\theta )\Big ) \bigg ) \end{aligned}$$

where $\varGamma$ is the fundamental solution of the time integrated FP equation centered around $(\mathbf {x'},\theta ')$ represented as:

3 $$\begin{aligned} \varGamma \Big ( (\mathbf {x},\theta ),(\mathbf {x'},\theta ') \Big ) = R^\mathbf {D}_\alpha \left( \mathbf {R}^\mathrm{T}_\theta (\mathbf {x-x'}),\theta -\theta '\right) \end{aligned}$$

with the resolvent kernel $R^\mathbf {D}_\alpha$ obtained by integrating Green’s function $K_t^\mathbf {D}: SE(2) \rightarrow {\mathbb {R}}^+$ as:

4 $$\begin{aligned} R^\mathbf {D}_\alpha (\mathbf {x},\theta ) = \alpha \int _0^\infty K_t^\mathbf {D}(\mathbf {x},\theta )\mathrm {e}^{-\alpha t}\mathrm {d}t \end{aligned}$$

which is the solution of the following PDE:

5 $$\begin{aligned} \left( \cos \theta \partial _x + \sin \theta \partial _y-\mathbf {D}\partial _\theta ^2-\alpha I\right) R^\mathbf {D}_\alpha = \alpha \delta _e. \end{aligned}$$

**Fig. 3:**
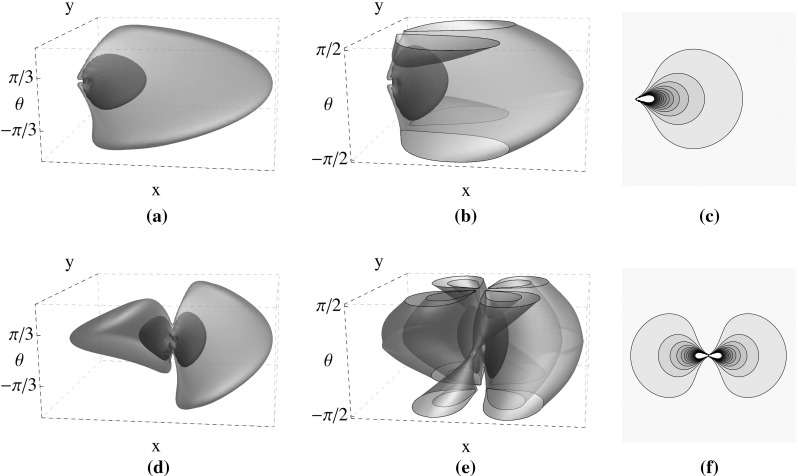
**a** Forward kernel in ${\mathbb {R}}^2\times S^1$, **b** $\pi -$shifted forward kernel in $\mathbb {R}^2\times \textit{P}^1$, **c** *xy*-marginal (obtained by integration over $\theta$) of the forward kernel, **d** forward–backward kernel in ${\mathbb {R}}^2\times S^1$, **e** $k^\mathrm{prob}$ in ${\mathbb {R}}^2\times P^1$, and **f** *xy*-marginal of $k^\mathrm{prob}$

In the above equations, $R^\mathrm{T}_\theta$ is the transpose of $R_\theta$ as a 2D counter-clockwise rotation matrix. In a Markov process traveling time is memoryless. The only continuous memoryless distribution is the negatively exponential distribution $T \sim NE(\alpha )$ with expected value $E(T)=\alpha ^{-1}$. As $P(T=t)=\alpha e^{-\alpha t}$, parameter $\alpha$ plays a role of a decay rate. Moreover, $\delta _e$ is the initial condition equals to $\delta _e =\delta _0^x \otimes \delta _0^y \otimes \delta _0^\theta$ ,where $\otimes$ denotes the tensor product in distributional sense [see  Zhang et al. (2016b) for detailed explanations]. Note that this PDE is defined on ${\mathbb {R}}^2\times S^1$ and not on ${\mathbb {R}}^2\times P^1$ as the first-order part flips when applying $\theta \rightarrow \theta +\pi$. Therefore, in Eq. 2, besides a $\pi$-shift, we need inversion invariance yielding a double symmetric kernel (see Fig. 3).

In this work the numerical solution has been created using the Fourier-based technique (Duits and Almsick 2008), because it is not only the best approximation to the exact solution, but also computationally the least expensive one compared to the other solutions (Zhang et al. 2016b). Referring to (Fig.13 Zhang et al. 2016b), the slight spatial blurring with $0<s\ll 1$ corresponds to a one-pixel bin used in statistical kernel. So the exact probability kernel (with small *s*) is considered with the same resolution as the statistical kernel. The final probability kernel and the statistical data-driven kernels are compared in the spatial domain. The key parameters in creating the probability kernel are $\alpha$ and $D_{33}=\sigma ^2/2$, which determine the expected life time of the resolvent kernel ($E(T)=1/\alpha$) and the diffusion matrix ($D = \text {diag}\{0,0,D_{33}\}$), respectively (see Zhang et al. (2016b) for more details). To have uniform notations in the rest of the article, we assume the following relations hold for both probabilistic and statistical kernels:

6 $$\begin{aligned}&k^\mathrm{{stat}} (h, g) =k^\mathrm{{stat}}( e, h^{-1}g) = K^\mathrm{{stat}}(h^{-1}g)\nonumber \\&\quad k^\mathrm{{prob}}(h,g) =k^\mathrm{{prob}}(e,h^{-1}g)=K^\mathrm{{prob}}(h^{-1}g) \end{aligned}$$

where $$g,h\in L_i$$ for $$i=1,\dots ,n$$.

As a side note, the exact non-symmetrized kernel on ${\mathbb {R}}^2 \rtimes P^1$ is given by:

7 $$\begin{aligned} R_{\alpha }^{{\mathbb {R}}^{2} \rtimes P^{1}}(\mathbf {x},\theta )= \left( \mathcal {F}^{-1}_{{\mathbb {R}}^2}[ \mathbf {\omega } \mapsto \sum \limits _{n \in \mathbb {Z}} \hat{R}_\alpha ^{\mathbf {D},\mathbf {a},\infty }(\mathbf {\omega },\theta + n \pi )] \right) (\mathbf {x})\nonumber \\ \end{aligned}$$

where $\hat{R}_\alpha ^{\mathbf {D},\mathbf {a},\infty }$ is expressed in two Mathieu functions in [Eq. 5.5, Zhang et al. (2016b)]. Alternatively, one may take the simplified exact solution on ${\mathbb {R}}^2\times S^1$ (Eq. 5.11 Zhang et al. 2016b) and consider only the first and third terms in Eq. 2.

## Experiments

In this section, first the datasets and the parameters used for finding the data-driven kernels are introduced. Then the results of the comparison of the data-driven kernels against each other and comparison of these statistical kernels with the probability kernels are presented. At the end, the application of the data-driven kernel in retrieving vessel connections is explained.

### Materials

**Fig. 4:**
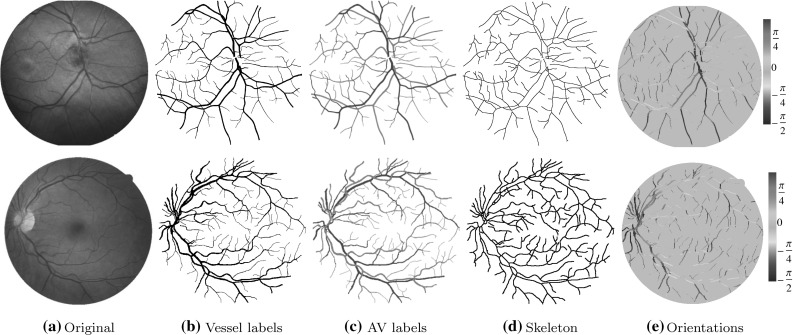
Two sample images from the IOSTAR (*top row*) and the DRIVE (*bottom row*) datasets. **a** The original images, **b** the vessel and **c** the AV ground truth images (artery in *red* and vein in *blue*), **d** the vessel skeleton and **e** the color-coded orientation maps

Two retinal datasets have been used in this study. The public DRIVE dataset (Staal et al. 2004) including 40 color fundus images taken with a Canon CR5 non-mydriatic 3CCD camera, with a resolution of $565 \times 584$, a pixel size of $25\,\upmu \mathrm{{m/px}}$ and a field of view of $45^\circ$. The second dataset is the public IOSTAR dataset^1^ (Abbasi-Sureshjani et al. 2015) including 24 images taken with a scanning laser camera (SLO) with a resolution of $1024 \times 1024$, a pixel size of $14\,\upmu \mathrm{{m/px}}$ and a field of view of $45^\circ$. The vessel ground truth and the AV labels are available for both datasets. Figure 4 shows two sample images (4a) from these two datasets together with their vessel (4b) and AV ground truth images (4c). The skeletons extracted from the vessel ground truth images are also presented there (4d). The color-coded images in the last column (4e) show the dominant angle at each pixel location (see Step 3 of Algorithm 1).

### The statistical kernels

The statistical kernel as explained in Sect. 2.1 is calculated for both datasets. The number of discrete orientations used is $n_\theta =16$, and *d* is set to 65. For each image both the full vasculature ground truth and the AV-separated ground truth images have been used and at the end, two different 3D histograms ($K^\mathrm{{stat}}$) are obtained per dataset. The histograms extracted directly from the full vasculature network are called $K^\mathrm{{stat}}_\mathrm{{DR}}$ and $K^\mathrm{{stat}}_\mathrm{{IO}}$ and the ones obtained from the AV-separated datasets are called $K^\mathrm{{stat}}_\mathrm{{DR-AV}}$ and $K^\mathrm{{stat}}_\mathrm{{IO-AV}}$. *DR* stands for the DRIVE, $\mathrm{IO}$ stands for the IOSTAR dataset, and *AV* stands for AV-separated.

**Fig. 5:**
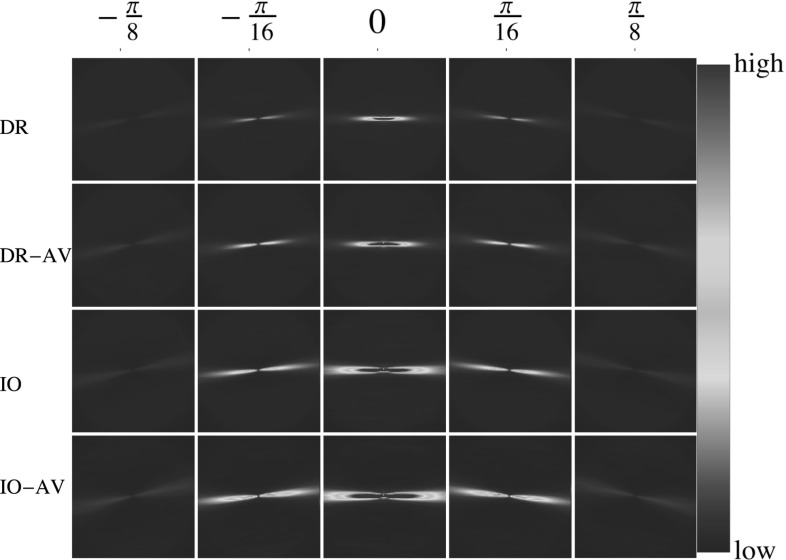
The statistical kernels obtained in Step 8 of Algorithm 1 for each dataset. The value of $\theta$ is shown for each column, and the values of all figures are clipped between 0 to 0.2 of the maximum value of the $K^\mathrm{{stat}}_\mathrm{{DR}}$

**Fig. 6:**
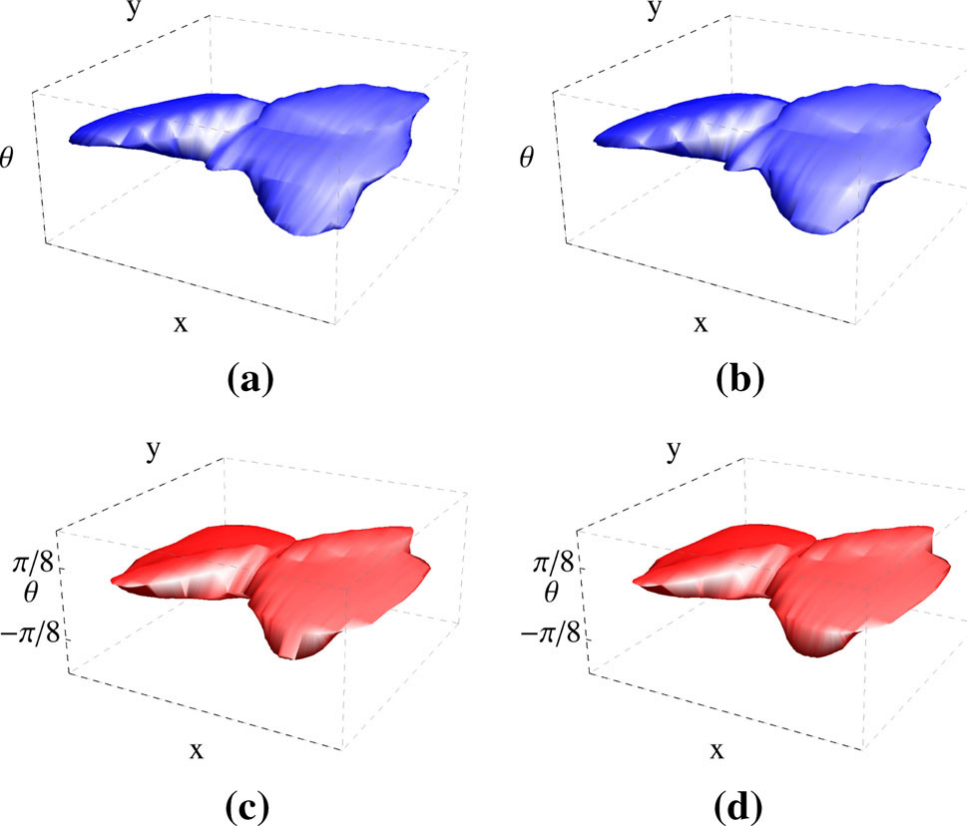
The level sets of the **a** $K^\mathrm{{stat}}_\mathrm{{DR}}$ and **b** $K^\mathrm{{stat}}_\mathrm{{DR-AV}}$ shown at 0.0125 of the maximum value of the $K^\mathrm{{stat}}_\mathrm{{DR}}$, and the levels sets of the **c** $K^\mathrm{{stat}}_\mathrm{{IO}}$ and **d** $K^\mathrm{{stat}}_\mathrm{{IO-AV}}$ shown at 0.025 of the maximum value of the $K^\mathrm{{stat}}_\mathrm{{IO}}$

Two different visualizations of the final statistical kernels are shown in Figs. 5 and 6. The rows in Fig. 5 from top to bottom represent the $K^\mathrm{{stat}}_\mathrm{{DR}}$, $K^\mathrm{{stat}}_\mathrm{{DR-AV}}$, $K^\mathrm{{stat}}_\mathrm{{IO}}$ and $K^\mathrm{{stat}}_\mathrm{{IO-AV}}$, respectively. Each square has the dimension of $131\times 131$, and it depicts the value of the kernel at fixed relative orientation ($K^\mathrm{{stat}}(\mathbf {x},\theta _c),~\theta _c\in \{\pm \pi /8,\pm \pi /16,0\}$). The kernel values of only five orientations are depicted as the information at other angles is very small. As seen in these figures, the maximum values of the statistical kernels occur at small orientation differences, i.e., two aligned lines are more probable to appear in the images. This probability decreases when the orientation differences increase. Comparing these four histograms qualitatively, the statistical kernels of the DRIVE dataset are a bit less elongated compared to the ones of the IOSTAR datasets. Moreover, the separation of the lines of arteries and veins from each other results in less noisy histograms for both datasets; however, the difference is very small. Figure 6 visualizes the isosurfaces of these four data-driven kernels, which shows their high similarity.

These data-driven kernels are compared with each other quantitatively, and their mutual differences are obtained as the absolute $\text {error}=\parallel K^\mathrm{{stat}}_1 - K^\mathrm{{stat}}_2 \parallel _{l_2}$, where $K^\mathrm{{stat}}_1$ and $K^\mathrm{{stat}}_2$ are the two statistical kernels in comparison. These least square errors are presented in Table 1 for each pair of kernels. Based on these quantitative results, their differences are very small. Therefore, it is possible to use them interchangeably, regardless of the dataset or the ground truth used for obtaining them.

**Table 1:** The least square errors obtained by comparing each pair of the statistical kernels

$K^\mathrm{{stat}}_1$	$K^\mathrm{{stat}}_2$	$\mathrm{Error}(\%)$
$\mathrm {DR}$	*IO*	1.14
$\mathrm{{DR}}$	$\mathrm{{DR-AV}}$	0.51
$\mathrm{{DR}}$	$\mathrm{{IO-AV}}$	2.14
$\mathrm{{IO}}$	$\mathrm{{IO-AV}}$	1.01
$\mathrm{{IO}}$	$\mathrm{{DR-AV}}$	0.69
$\mathrm{{DR-AV}}$	$\mathrm{{IO-AV}}$	1.66

### Comparison to the probability model

In this section we find the best approximations of the statistical kernels by comparing them against various probability kernels (obtained using Eq. 2) with different parameters. The kernels that result in the least square errors are considered as the best approximations. In our experiments, the parameter $\alpha$ takes 50 different values from 0.00001 to 0.01 and the parameter $D_{33}$ takes 100 values from 0.000001 to 0.005. These ranges are determined heuristically. The minimum errors and the corresponding parameters for each kernel are presented in Table 2. Based on these results, the errors are very small for all the kernels. The largest error is related to the $K^\mathrm{{stat}}_\mathrm{{IO-AV}}$, which is close to $1\%$.

**Table 2:** The least square errors and the corresponding parameters resulting in these errors between the statistical and probability kernels

$K^\mathrm{{stat}}$	Error ($\%$)	$\alpha$	$D_{33}$	$\sigma$
$\mathrm{{DR}}$	0.3275	0.0024	0.00170	0.0583
$\mathrm{{DR-AV}}$	0.55	0.0048	0.00210	0.0648
$\mathrm{{IO}}$	0.8109	0.0080	0.00130	0.0509
$\mathrm{{IO-AV}}$	1.04	0.0098	0.00085	0.0412

**Fig. 7:**
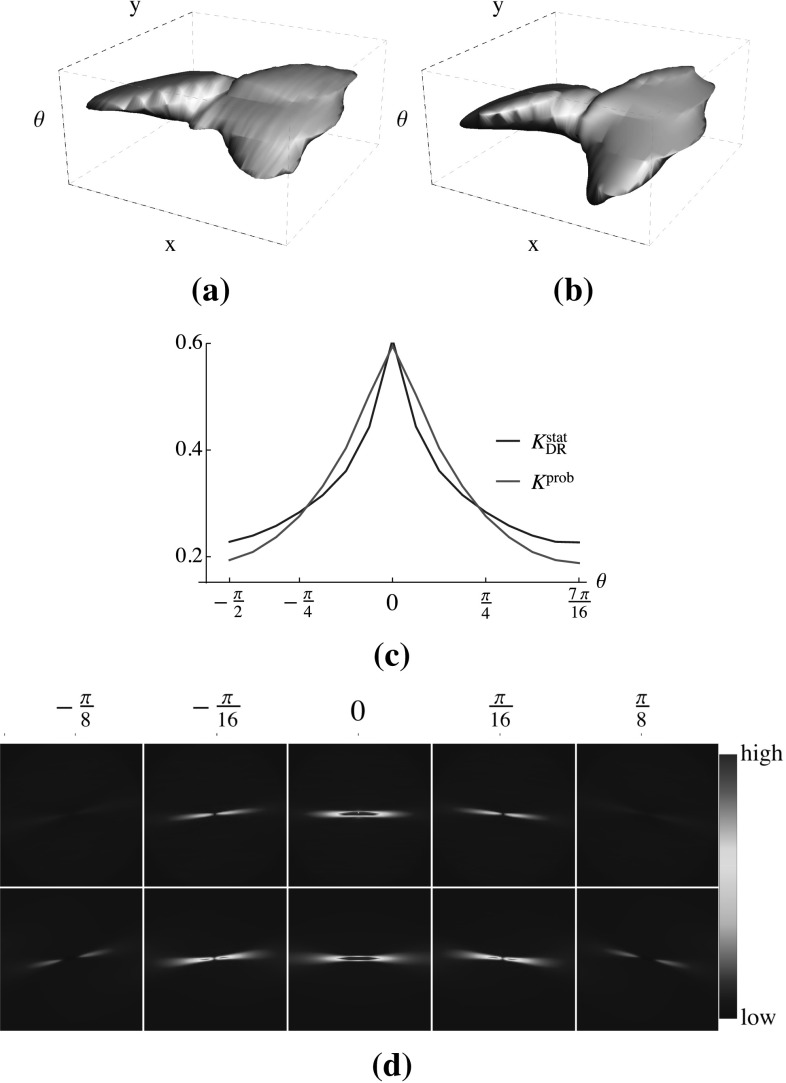
Comparison between the $K^\mathrm{{stat}}_\mathrm{{DR}}$ and its best approximating kernel $K^\mathrm{{prob}}_\mathrm{{DR}}$: the level sets at 0.012 of the maximum value of **a** the statistical and **b** the probability kernels, **c** the summation of the values of the two kernels over the spatial dimension at different angles, and **d** the line distribution at five different orientations $\theta \in \{\pm \pi /8,\pm \pi /16,0\}$ in the data-driven (*top row*) and the probability kernel (*bottom row*)

A sample qualitative comparison of these two types of kernels is shown in Fig. 7. In addition to very similar profiles of the two kernels in Fig. 7a–c the summations of the line distribution over the spatial dimension also matches the information density of the probability kernel at every $\theta$ layer (Fig. 7c). In both kernels, the maximum value appears at $\theta =0$ as expected.

### Application in retinal image analysis

In this section, the application of the statistical model of the cortical connectivity pattern in identifying vessel connections in retinal images is explained.

In this method the vessel connections are retrieved from image segmentations (not necessarily centerlines). So an initial segmentation of the image *I* (using the aforementioned segmentation techniques proposed in the literature) is required. The binary image representing the segmentation is called $I_\mathrm{s}$. Repeating the steps as the ones proposed in Algorithm 1 (Steps 2, 3 and 4) the image is lifted ($U_\mathrm{I}$), dominant orientations ($\theta _m$) are obtained, and the set of interest points is created as:

$$L= \{(\mathbf {x},\theta _{m}(\mathbf {x}))~|~I_{s}(\mathbf {x}) = 1\}.$$

Considering $m = |L|$, an $m\times m$ affinity matrix (*A*) is created to take into account the connection probability between each pair of points in set *L* and is later aggregated with another affinity matrix ($\tilde{A}$) representing the similarities of the corresponding vessel intensities. So for each pair of $(\mathbf {x}_i,\theta _m(\mathbf {x}_i))$ and $(\mathbf {x}_j,\theta _m(\mathbf {x}_j))\in L$:

8 $$\begin{aligned} A_\mathrm{{final}}(\mathbf {x}_i,\mathbf {x}_j)= & {} A(\mathbf {x}_i,\mathbf {x}_j) \tilde{A}(\mathbf {x}_i,\mathbf {x}_j)\nonumber \\= & {} k^\mathrm{{stat}}((\mathbf {x}_i,\theta _m(\mathbf {x}_i)),(\mathbf {x}_j,\theta _m(\mathbf {x}_j)) \nonumber \\&\times \, G_{\sigma _\mathrm{{int}}}(I_n(\mathbf {x}_i)-I_n(\mathbf {x}_j)),\nonumber \\ \quad i,j= & {} 1,\dots ,m. \end{aligned}$$

where $G_{\sigma _\mathrm{{int}}}(x)$ is the normalized Gaussian kernel with the standard deviation of $\sigma _\mathrm{{int}}$ and $I_n$ is the image intensity in normalized green channel. Here we normalized luminosity and contrast using the method by Foracchia et al. (2005). The final affinity matrix is analyzed using the self-tuning spectral clustering technique (Zelnik-Manor and Perona 2004), which identifies the salient groups in the image automatically.

**Fig. 8:**
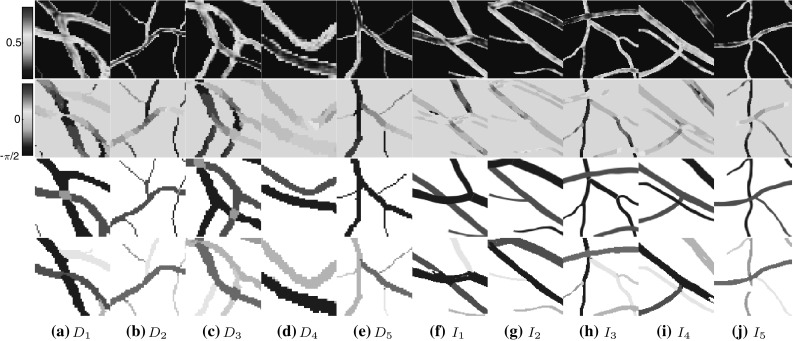
Some example retinal patches selected from the DRIVE ($D_1$ – $D_5$) and the IOSTAR ($I_1$ – $I_5$). The first three rows from top to bottom indicate the intensity, main orientations and the AV labels of the vessels. The last row represents the final clustering results after removing the small groups (noise). Detected clusters are shown in different colors

As discussed before, the differences between the kernels obtained from the same datasets are very small and they can be used interchangeably. Hence, for analyzing the retinal image patches taken from the DRIVE and IOSTAR datasets we only use the $K^\mathrm{{stat}}_\mathrm{{DR-AV}}$ and the $K^\mathrm{{stat}}_\mathrm{{IO-AV}}$ kernels, respectively. For testing the method, several patches with the sizes of $51\times 51$ and $101\times 101$ have been selected around junctions from the DRIVE and IOSTAR datasets, respectively. A vessel segmentation by Abbasi-Sureshjani et al. (2015) developed for both color RGB images and SLO images is applied on these patches. After segmenting the images, the positions, orientations and normalized intensities for the vessel pixels are extracted.

Figure 8 shows five image patches for each dataset. The first five patches ($D_1$ – $D_5$) belong to the DRIVE, and the second five patches ($I_1$ – $I_5$) are selected from the IOSTAR datasets. For each patch, from top to bottom the vessel intensities, the color-coded orientation maps [representing the values of $\theta _m(\mathbf {x})$], the AV labels and finally the clustering results have been presented. As seen in this figure, the detected final perceptual units (shown in different colors) correspond to the separate blood vessels existing in the image patch. These patches have been selected in a way to represent several complex topological structures of the blood vessels, varying the number of the vessel bifurcations and crossings and existence of parallel or curved vessels. For all the experiments the parameter $\sigma _\mathrm{{int}}$ was set to 0.2. Despite the complexity of the structures, using orientation and intensity features for determining the contextual connections among pixels individuates well the blood vessels from each other. Using the feature of intensity helps in the cases where there is an abrupt change in the orientation but not the intensity, e.g., in $D_3$ and $D_4$. Moreover, the presence of disconnections (e.g., in $I_5$) does not affect the correct grouping. In addition to these patches, 20 more patches per dataset have been analyzed. For all these cases, the method is successful in correctly grouping the vessel pixels. The limitation arises when both the feature of intensity and orientation of a vessel are very noisy or change suddenly. In these cases, the vessel splits into smaller clusters. Involving additional contextual information such as curvature or scale may help in resolving this problem as proposed by Abbasi-Sureshjani et al. (2016).

## Conclusion

In this work, we exploit the relation between the statistical co-occurrences of line elements in natural images and the high-level task of contour grouping in our brain, and use it in the retinal image processing for contour completion. Firstly, the steps for obtaining the rotation and translation-invariant line statistics from different retinal image datasets are explained. Secondly, their relation to the symmetrized probability kernel of the direction process on the projective line bundle modeling the cortical connectivity is investigated. The results reveal their remarkable high similarity. However, in edge co-occurrences (rather than line co-occurrences) of natural images (e.g., Sanguinetti et al. 2010) the shapes seem to resemble the shape of the hypo-elliptic heat kernels (without convection) in ${\mathbb {R}}^2\times P^1$, but the relation needs further investigation. In addition, all the statistical kernels obtained from different datasets are compared with each other quantitatively and qualitatively. The obtained results indicate their high similarity and reproducibility despite the differences in the datasets and the setups used.

Furthermore, the statistical kernels were used directly to retrieve the individual vessels from segmented retinal images. The successful results show that Mumford’s direction process is a very good model for centerlines of vessels, and together with the Lie group theory, the proposed connectivity analysis technique is useful for retinal image analysis. Using the data-driven kernel (that does not need parameter tuning) in addition to adding the automatic self-tuning spectral clustering technique, forms a robust and fully automatic connectivity analysis technique. This provides an effective solution for challenging situations in which most of the methods fail (but our visual perception succeeds) because of non-perfect imaging conditions, interruptions or occlusions.

The method presented here for extracting line co-occurrences from retinal images and the improved connectivity analysis approach can be extended to a rich number of other application areas which contain curvilinear structures such as corneal never fibers, plant roots and road networks. For each dataset, it is possible to learn the connectivity kernel and use it directly for similar images.

Since the visual cortex deploys additional contextual information and its receptive fields are not only sensitive to orientation, but also other information (such as scale and curvature), one potential extension of the method is to use this additional information in deriving the line statistics and creating higher-order kernels.

## Footnotes

1 Available at: http://www.retinacheck.org/datasets.
